# Link between Diabetes and Alzheimer’s Disease Due to the Shared Amyloid Aggregation and Deposition Involving Both Neurodegenerative Changes and Neurovascular Damages

**DOI:** 10.3390/jcm9061713

**Published:** 2020-06-03

**Authors:** Gabriela Dumitrita Stanciu, Veronica Bild, Daniela Carmen Ababei, Razvan Nicolae Rusu, Alina Cobzaru, Luminita Paduraru, Delia Bulea

**Affiliations:** 1 Center for Advanced Research and Development in Experimental Medicine (CEMEX), Grigore T. Popa” University of Medicine and Pharmacy, 16 Universitatii Street, 700115 Iasi, Romania; gabriela-dumitrita.s@umfiasi.ro; 2 Pharmacodynamics and Clinical Pharmacy Department, Grigore T. Popa” University of Medicine and Pharmacy, 16 Universitatii Street, 700115 Iasi, Romania; dana.ababei@gmail.com (D.C.A.); razvan.nicolae.rusu@gmail.com (R.N.R.); alimoraru23@yahoo.com (A.C.); dbulea@yahoo.com (D.B.); 3 Department Mother & Child Care, Division Neonatology, Grigore T. Popa” University of Medicine and Pharmacy, 16 Universitatii Street, 700115 Iasi, Romania

**Keywords:** diabetes, Alzheimer’s disease, amyloidogenic diseases, islet amyloid polypeptide, amyloid-β peptide, neurodegenerative changes, neurovascular damages, in vivo models

## Abstract

Diabetes and Alzheimer’s disease are two highly prevalent diseases among the aging population and have become major public health concerns in the 21st century, with a significant risk to each other. Both of these diseases are increasingly recognized to be multifactorial conditions. The terms “diabetes type 3” or “brain diabetes” have been proposed in recent years to provide a complete view of the potential common pathogenic mechanisms between these diseases. While insulin resistance or deficiency remains the salient hallmarks of diabetes, cognitive decline and non-cognitive abnormalities such as impairments in visuospatial function, attention, cognitive flexibility, and psychomotor speed are also present. Furthermore, amyloid aggregation and deposition may also be drivers for diabetes pathology. Here, we offer a brief appraisal of social impact and economic burden of these chronic diseases and provide insight into amyloidogenesis through considering recent advances of amyloid-β aggregates on diabetes pathology and islet amyloid polypeptide on Alzheimer’s disease. Exploring the detailed knowledge of molecular interaction between these two amyloidogenic proteins opens new opportunities for therapies and biomarker development.

## 1. Socio-Economic Burden of Diabetes and Alzheimer’s Disease

**Figure 1:**
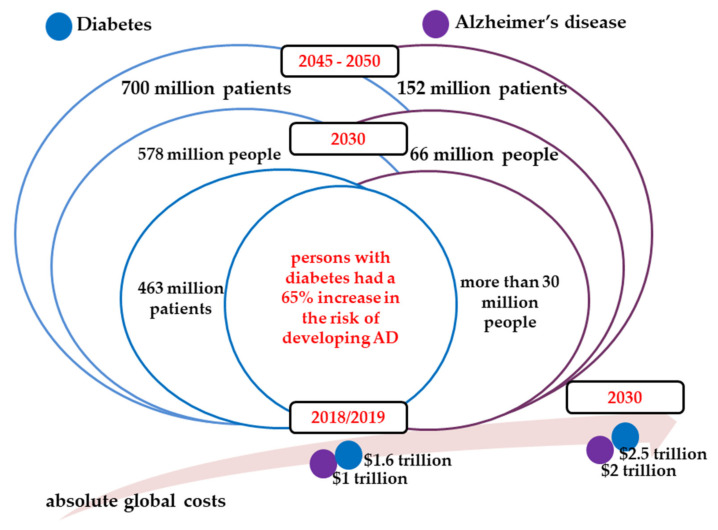
Global diabetes and Alzheimer’s disease (AD) prevalence estimates and economic burden for 2018/2019 and projections to 2030 and 2050.

Diabetes, both type 1 and 2, and Alzheimer’s disease (AD) are vastly prevalent conditions among the elderly population, with a multifactorial nature involving various biological mechanisms that continue to be important, as well as growing health challenges worldwide [1]. Due to its continuous rise of prevalence in developed and developing countries over recent years, diabetes is considered a global key health priority. Estimates show that in most countries the burden put out by the disease is increasing, this being evidence of an increase of risk in the population [2]. The number of people living with diabetes tripled between 1990 and 2010, whilst the number of new cases annually doubled [3]. There are approximately 463 million patients worldwide (9.3%) affected by the disease rising to 578 million (10.2%) by 2030 and 700 million by 2045 (10.9%) [4]. The absolute global cost of diabetic patients in 2018 has been estimated at $ 1.6 trillion, which corresponds to a gross domestic product of 1.8%, and this figure will increase to $ 2.5 trillion annually by 2030 (Figure 1) [5]. 

AD is an irreversible, progressive form of dementia culminating in gradual memory loss, various cognitive impairments, and intellectual incapacities that interfere with quality of life [6]. In an attempt to update the guidelines toward a biological definition of Alzheimer’s disease, the National Institute on Aging and Alzheimer’s Association Research Framework has generated distinct diagnostic recommendations for the preclinical, mild cognitive impairment, and dementia stages of AD. It has become clearer that AD is both a pathophysiological condition and a clinical entity at the same time; specifically, Alzheimer’s disease begins decades before the onset of the earliest clinical manifestations, and Alzheimer’s dementia is the actual last condition in which AD culminates [7]. Around the world, nearly 50 million people were afflicted with dementia in 2018, and AD accounted for 60% to 80% of these cases. In the coming three decades, this number is projected to more than triple, at 152 million. The total estimated overall cost for the care of AD patients in 2018 was estimated at $1 trillion, which corresponds to a gross domestic product of 1.2%, and this figure will increase to $2 trillion annually by 2030—costs that can weaken social and economic development and could overwhelm health and social services (Figure 1) [1,6]. Moreover, these chronic conditions are no longer a problem only for high-income countries. More than two-thirds of all diabetes and AD patients live in the United States of America, India, Brazil, and China, and this percentage will increase by 2050 [4,8].

The high individual, familial, social, and financial burden of diabetes and AD requires bold and critical plans. As a result, most states and many international organizations have elaborated strategies and analytical reports to solve the problem [4], and research based on mathematical and animal models or on qualified estimates are trying to predict the development of diabetes in relation to AD. Table 1 shows the current pharmacotherapeutic approaches in diabetes and AD. Existing results do not support the repurposing of diabetes therapies for dementia risk decrease or management. Studies in the field of diabetes and AD are active, and additional data are required before final conclusions can be drawn.

In the present paper, we sought to offer a brief overview of social impact and economic burden of these chronic diseases and provide insight into amyloidogenesis in light of recent advances of amyloid-β aggregates on diabetes pathology and islet amyloid polypeptide on AD. Exploring the detailed knowledge of molecular interaction between these two amyloidogenic proteins opens new opportunities for therapies and biomarker development that can prevent or reduce the occurrence of diabetes, as well as AD.

## 2. Amyloid Formation as a Common Pathological Feature in both Diabetes and Alzheimer’s Disease

### 2.1. Relations between Diabetes and Alzheimer’s Disease

Since the beginning of the 21st century, multiple studies have highlighted the potential links between AD and abnormalities of insulin signaling related with diabetes. The link seems to be so robust that AD is frequently considered a neuroendocrine disorder referred to as “diabetes type 3” or “brain diabetes” [9]. It was found that diabetic patients had a 65% increase in the risk of developing AD and display lower basal cognitive skills including learning, memory, and perceptual speed [10]. Somewhat surprisingly, AD and diabetes share several risk factors, such as, higher cholesterol, degeneration, beta-amyloid deposition [11], oxidative stress, inflammation cardiovascular disorders, dysmetabolism syndrome [12], glycogen synthesis kinase 3, τ protein phosphorylation [13], apolipoprotein E4 [14], and apoptosis. In addition, reports have revealed that insulin contributes substantially to neurological function by stimulating the expression of the enzyme that controls the acetylcholine synthesis. Consequently, suboptimal insulin values as well as low insulin receptor sensitivity could eventually contribute to a reduction in acetylcholine, which leads to a likely biochemical link between diabetes and AD [8,11,14]. Such associations can reveal a direct effect of uncontrolled hyperglycaemia on neurodegenerative changes in the brain. This can also be due to diabetes-related comorbidities (e.g., hypertension, dyslipidaemia), or can be an effect of hyperinsulinemia or impaired insulin response [15,16,17]. Hyperinsulinemia, which comes with diabetes type 2, is a factor that links AD and diabetes, being related to amyloid-beta peptide (Aβ) accumulation in the brain. By competing for the insulin-degrading enzyme, it disrupts brain Aβ clearance. In the pathogenesis of diabetes, receptors for advanced glycation end-products are also involved. These receptors can be present in cells associated with senile plaques, with neurofibrillary tangles being a cell surface receptor for Aβ. Another predisposing diabetes factor is the excess of adipose tissue, through production of adipocytes critical to metabolism and cytokines involved in the inflammatory process. Adiponectin, leptin, resistin, tumor necrosis factor alpha (TNF-α) and interleukin 6 (IL-6) are produced, being tied to insulin resistance and hyperinsulinemia, thus directly or indirectly affecting AD risk [18]. Using brain imaging, subjects with diabetes have hippocampal and amygdala atrophy when compared to nondiabetic subjects. The hippocampus and amygdala are responsible for functions such as behavior and memory and are also atrophied in AD. Brain microvascular lesions and extensive amyloid plaque burden coexist in brains of patients with diabetes and dementia, as shown in post-mortem studies, with these characteristics being present in AD as well [19]. Reduced morbidities and premature deaths will lead to a longer life for patients with diabetes, but this will increase costs because of the necessary management of the multiple chronic conditions the disease is associated with. Population with undiagnosed and untreated diabetes mellitus type 2 are at a superior risk of complications compared with those who are receiving treatment. In many cases, the onset of diabetes mellitus often happens years before the concrete diagnosis, being considered that approximately 174.8 million cases were undiagnosed [20].

### 2.2. Amyloid Formation and Deposition Involving both Neurodegenerative Changes and Neurovascular Damage

**Figure 2:**
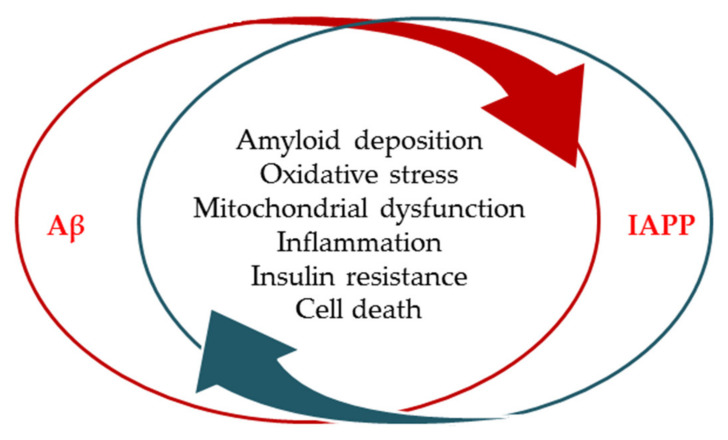
Schematic representation of the correlations between islet amyloid polypeptide of diabetes and amyloid-β peptide from Alzheimer’s disease; conditions characterized by cell loss and abnormal of Aβ, tau, and amylin deposition. These aggregates could stimulate amyloid formation and deposition by cross-seeding in pancreatic cells and neurons. The presence of tau and amylin aggregates supports the aggregation of beta-amyloid deposits, which leads to oxidative stress, mitochondrial abnormalities, inflammation, insulin resistance, and ultimately death of cells. Aβ, amyloid-β peptide; IAPP, islet amyloid polypeptide.

The pathological process of amyloidogenesis, in which proteins or peptides self-assemble naturally into higher order structures, called oligomers, protofibrils, and mature amyloid fibrils, occurs in several diseases, including AD and diabetes. However, data highlighting whether these various forms of amyloid proteins interact or how the formation of these amyloid structures begins are very scarce [21,22]. Amyloids gather into aggregates forming Aβ oligomers and fibrils in AD and islet amyloid polypeptide (IAPP) or amylin in diabetes. IAPP is one of the most important β-cell secretory products of the pancreatic islets of Langerhans. With putative function, IAPP is a regulatory peptide both locally in the islets, where it inhibits insulin and glucagon secretion, and at distant targets. It has binding sites in the brain, possibly contributing to the regulation of satiety and inhibition of gastric emptying, and has also been described as having effects on several other organs over time. IAPP has been identified due to its ability to aggregate in the amyloid deposits of pancreatic islets, which are seen primarily in association with type 2 diabetes in humans and diabetes in several other mammalian species, especially monkeys and cats [23]. At present, it is increasingly accepted that there are close correlations between these two diseases by overlapping their pathology, sharing common complications including impaired carbohydrate metabolism, insulin resistance, oxidative stress, inflammatory response, mitochondrial dysfunction, and amyloidosis (Figure 2) [24]. 

Although several pathological processes appear to be shared in both diabetes and AD, the molecular mechanism underlying these conditions is not completely known [25]. Like Aβ deposition in AD, IAPP is also aggregated in people with diabetes to form pancreatic islet amyloid, and its detection in brain tissue has been linked with cognitive decline. Structurally, IAPP and human Aβ contain a total of 25% identical amino acid sequences that have high binding affinity, many biophysical and physiological properties, and which exert similar cytotoxic mechanisms when aggregated. Additionally, in patients with AD, IAPP deposits that contribute to the pathophysiology of the disease were found in the brain tissue [26].

IAPP is synthesized and secreted by β-cells of the pancreatic islets of Langerhans, together with insulin. Typically, IAPP is co-secreted with insulin at a molar ratio of 1:100 [27]. In β-cell secretory granules, IAPP is mainly located in the halo region, whereas insulin is stored in the dense core of β-cells in microcrystalline groups [28]. In humans, IAPP is secreted by hormone pluripotent endocrine stem cells in all stages of embryonic development. The first IAPP-positive cell appears after only 13 weeks of gestation. The gene encoding IAPP is located on chromosome number 12 [29]. IAPP is synthesized as a pre-pro form of IAPP that contains 89 amino acids. The 22 amino acid signal peptide is cleaved to give the 67 amino acid pro-form (pro-IAPP). Pro-IAPP is processed in the Golgi apparatus and in the insulin secretory granules [26,30,31]. The physiological roles of soluble IAPP in the body are not yet fully known. IAPP is a gluco-modulatory hormone that inhibits insulin secretion, delays gastric emptying inhibits appetite, and plays a role in regulating food intake and controlling body weight [31]. The IAPP belongs to the calcitonin family of hormones, presents the functions of “bone remodelling”, inhibits osteoclast activity, and enhances osteoblast proliferation [29]. Several studies have explored the mechanisms responsible for the formation of amyloid deposits, which explain the cytotoxic effects of IAPP. Amylin is a peptide with 37 amino acid residues. The sequence in the 20–29 regions determines its ability to form amyloid deposits. Humans, non-human primates, and cats form in vivo deposits of amyloid. It is a phenomenon not found in rats and mice [30], as there is a considerable difference in the amino acid sequence 20–29 in different species. In the cases of mice and rats, there are three proline residues in the 20–29 segments that modify the beta-sheet conformation required for IAPP aggregation. In humans, this segment, called the amyloidogenic region, does not contain proline residues and is essential for IAPP aggregation [26]. Human IAPP levels released from beta cells may be another important factor in the process of amylin aggregation [32,33]. Poor processing of pro-IAPP also plays a central role in the aggregation of IAPP and in the formation of amyloid deposition in vivo [30]. In order to form amyloid deposits, it is necessary to use different biological, chemical, or physical factors [31]. A still controversial issue is whether IAPP aggregation is initiated intracellularly or extracellularly. Identifying the initial site of aggregation is important in discovering drugs capable of crossing beta cell membranes and thus being able to inhibit the formation of initial IAPP aggregates. The formation of small aggregates in the intracellular space can lead to the death of beta cells. Small aggregates can be secreted into the extracellular zone where they can then act as seeds for further propagation of amyloid deposits into the extracellular space [26].

Pathologically, amyloid plaques representing extracellular accumulations of Aβ peptides and intracellular neurofibrillary agglomerations, composed of tau proteins associated with microtubules, are specific to AD [34]. The development of amyloid plaques is an early and invariable attribute of AD and the general opinion is that the accumulation of Aβ peptides is an event that triggers tau pathology, resulting in defective neuronal functioning and cell loss. In AD, autosomal dominant Aβ peptides accumulate due to mutations that cause their overproduction. The cause of elevated brain levels of Aβ peptides in AD is unclear, but most likely is due to defective clearance compared to overproduction. Aggregation of Aβ peptides is a main aspect in the pathogenesis of AD. While amyloid plaques are made of highly ordered fibrils of Aβ peptides, it appears that soluble oligomers of these peptides, probably in the form of small dimers, are much more pathogenic [22,35,36]. There is plenty of evidence to support the amyloid cascade hypothesis as the main hypothesis explaining the mechanisms leading to Alzheimer’s disease. This hypothesis states that Aβ, a proteolytic fragment of β-amyloid precursor protein (APP), plays a dominant role in pathogenesis. Aβ peptides are the major protein component of the neuritic plaques characteristic of AD—they are extracellular lesions composed of a central nucleus of aggregated Aβ peptides surrounded by dystrophic neurites, activated microglia, and reactive astrocytes [26,37,38]. It has been shown that aggregate forms of synthetic Aβ peptides can affect neuronal cell cultures in vitro. Recent research suggests that soluble oligomeric prefibrillar forms of Aβ (so-called diffusible Aβ derivatives (ADDLs) or protofibrils), rather than highly aggregated Aβ forms, may be neurotoxic and cause synaptic dysfunction [39]. The alleged sequence of major pathological processes leading to neurodegeneration in AD, consistent with the hypothesis of the amyloid cascade issued by Hardy and Selkoe [40], subsequently updated [41], includes:

▪ Misplaced mutations of the APP, presenilin−1 (PSEN−1), and or presenilin−2 (PSEN−2) genes that may result in increased production of Aβ42 peptides throughout life in the dominant forms of AD or,
▪ by impairing the Aβ peptide purification mechanisms that would favor the gradual increase of the Aβ42 peptide level in the brain in the case of non-dominant forms of AD.

Both processes lead to the accumulation of the Aβ42 peptide in the limbic system, as well as in the associated cortex that initially produced discrete effects on synaptic efficiency. Subsequently, these accumulations of peptides determine the formation of dispersed plaques that activate microglia and astrocytes and the appearance of the inflammatory effect, which alters neuronal homeostasis. Neuronal dysfunction and loss of neurons lead to imbalances of neurotransmitters that will generate dementia [40,41]. 

**Figure 3:**
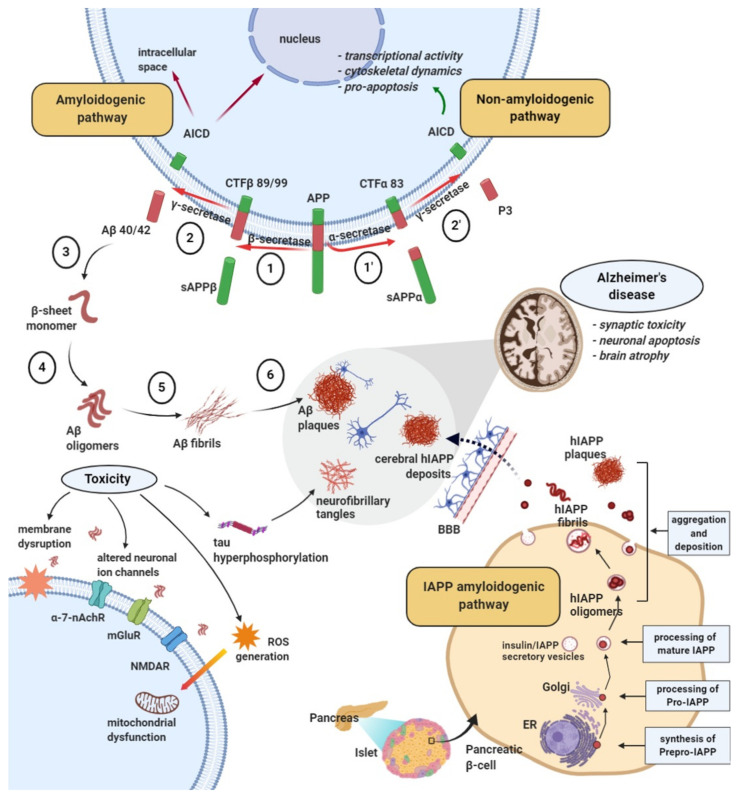
Amyloidogenic pathways of amyloid-beta peptide development in Alzheimer’s disease and islet amyloid polypeptide in diabetes. (1) Proteolytic cleavage of the transmembrane glycoprotein β-amyloid precursor protein (APP) by beta-secretase (amyloidogenic pathway) into the extracellular sAPPβ and the membrane β-C-terminal membrane-bound (CTF-β) 89/99 or (1′) by alpha secretase resulting into extracellular sAPPα and membrane CTF-α 83 fragment (non-amyloidogenic pathway). (2) Further cleavage of the CTF-β fragments at multiple sites by γ-secretase, resulting in the formation of extracellular Aβ 40–42 monomers and intracellular APP intracellular domain (AICD) that can translocate to the nucleus (2′) CTF-α cleavage by γ-secretase generating non-toxic extracellular P3 peptide and intracellular AICD. (3) Aβ conformational rearrangement from α-helical structure to β-sheet secondary structure monomers. (4) Assemblage of β-sheet Aβ monomers into soluble oligomers responsible for neuronal toxicity through mitochondrial dysfunction and membrane receptor binding. (5) Gradual aggregation of Aβ oligomers into insoluble amyloid fibrils and (6) deposits of amyloid senile plaques characteristic of AD. The initially 89 amino acid long islet amyloid polypeptide (IAPP) pre-pro peptides/along with the pro-insulin precursor/are synthesized in the endoplasmic reticulum of the pancreatic islet β-cells and then processed by enzymatic cleavage at both C-terminal and N-terminal to 67 amino acid pro-IAPP peptide. The pro-IAPP intermediate is subsequently cleaved by enzymes/prohormone convertase in the Golgi apparatus and in the secretory granules to form the 37 amino acid IAPP molecules. Mature IAPP stored together with insulin in the secretory granules may undergo misfolding processes, leading to potentially toxic intracellular and subsequent extracellular IAPP oligomers. Overexpression of the human IAPP molecules further induces IAPP aggregation into amyloid fibrils and plaques deposited intra- and extracellularly. IAPP oligomers may enter in the brain by crossing the blood–brain barrier, and IAPP deposits may contribute to AD pathology. Aβ, amyloid-β peptide; APP, amyloid precursor protein; AICD, cytoplasmic polypeptide; BACE1, beta-secretase 1; BBB, blood–brain barrier.

Starting from these subsequent stages that support the hypothesis of amyloid cascade, at present, there are a number of findings that would diminish its importance in AD as well as a series of counterarguments that advocate for the discrepancies [41,42]. In this regard, it was found that a large number of people had abundant Aβ deposits at the post-mortem autopsy but they were not clearly visible during their life with dementia, and a counter argument would be that these deposits are under the form of diffuse plaques, less neurotoxic up to a certain level [40,41]. The failures of several anti-amyloid immunotherapies have led many researchers to reject the hypothesis of amyloid in AD, but the significant efficacy in the phase 3 EMERGE study of an anti-amyloid antibody (aducanumab) is an important validation of amyloid as a therapeutic target [43]. APP is a type I transmembrane protein discovered 30 years ago with a role in the pathogenesis of AD but that also contributes to regulating important physiological functions such as central and peripheral nervous system development including synaptic plasticity and cognition processes (learning and memory) [44], encoded by the APP gene on chromosome 21 [25]. The APP695 isoform is the predominant form in the central nervous system [26]. To release Aβ from the APP, two cleavages are required, one in the extracellular area (cleavage by β-secretase) and the other in the transmembrane region (cleavage by γ-secretase) [45,46]. APP is first cleaved within the lumen by β or α-secretase, resulting in the almost complete “cutting” of the ectodomain and the generation of β- or α-C-terminal membrane-bound (β- or α-CTF) fragments, respectively. These fragments are subsequently cleaved within the transmembrane domain by γ-secretase with the release of peptides Aβ (4 kDa) and p3 (3 kDa), respectively, into extracellular media. Additionally, cleavage by γ-secretase generates a cytoplasmic polypeptide called AICD (APP intracellular domain) (Figure 3). 

Non-amyloidogenic processing of APP refers to the sequential processing of APP by membrane-bound α- and γ-secretases. A-secretases were the first proteolytic enzymes identified as cleaving APP within the Aβ domain, thus preventing the generation of intact Aβ peptides. Further processing of truncated Aβ (p3) and AICD peptides is not fully known. Amyloidogenic processing of APP is accomplished by the sequential action of membrane-bound β- and γ-secretases [46]. Main neuronal β-secretase is a transmembrane aspartyl protease called BACE1 (β-site APP-cleaving enzyme 1). APP cleavage by BACE1 generates N-terminal fragments of Aβ [47].

Subsequent studies have indicated that γ-secretase, a protein complex, performs enzymatic cleavage at multiple locations within the transmembrane domain of APP, generating Aβ peptides with variable length from 38 to 43 residues. Almost 90% of the Aβ peptides are generated at residue 40 (Aβ40), while less than 10% are generated at residue 42 (Aβ42). Smaller amounts of shorter peptides (Aβ37 or Aβ38) have also been identified. APP mutations identified in familial AD beyond the C terminal of the Aβ domain lead to elevated levels of Aβ42 [38,39,48]. Mutations in PSEN−1 and PSEN−2 associated with familial AD influence APP cleavage by γ-secretase through an unknown mechanism that variably affects the specificity of the cleavage site, generally favoring cleavage at position 42 over position 40, thus increasing the Aβ42/40 ratio. In the normal state, APP is initially cleaved by α-secretase with the generation of sAPPα and a C83 carboxy-terminal fragment. The presence of sAPPα is associated with normal synaptic signaling and results in synaptic plasticity, learning and memory, emotional behavior, and neuronal survival. In pathological state, APP is sequentially cleaved by β-secretase and γ-secretase with the release of an extracellular fragment called Aβ40–42. This neurotoxic fragment frequently aggregates with Aβ40–42 oligomerization and amyloid plaque formation. Aβ40–42 aggregates cause negative effects such as ion channel blockage and impaired calcium homeostasis, leading to mitochondrial oxidative stress, impaired energy metabolism, abnormal glucose regulation, and, finally, nerve cell death [21,34,46,49], as can be seen in Figure 3. 

### 2.3. Evidence from the Shared Pathological Traits

In recent decades, many clinical studies have shown that patients with diabetes perform less well in activities that involve cognitive processes such as memory, attention, information processing, and executive function. An important role in this regard was found by the cross-sectional clinical study based on the measurement of some cognition indicators affected in diabetes (UDES). In this study, 122 patients with diabetes mellitus aged 56–80 were submitted to brain magnetic resonance imaging (MRI), neurological and neuropsychological examinations, medication evaluation, and measurement of several clinical parameters. The impairment of the cognitive parameters could be caused by the cortical and subcortical atrophy as well as by the brain infarction revealed with the help of the brain MRI [26,50]. Diabetes mellitus is one of the risk factors for the pathogenesis of AD by impairment of insulin signaling and glucose metabolism, both centrally and peripherally [51]. 

The occurrence of islet amyloid in people with diabetes varies between almost 100% and 40% or even less [27]. Maloy et al. [52] evaluated the presence of amyloid deposits in the pancreas in diabetic and non-diabetic patients. It was identified in 59% of the diabetic cases and 12% of the non-diabetic cases. Diabetics treated with insulin had the highest prevalence (89%) and the most severe degree of pancreatic islet amyloidosis. There was an important correlation between the severity of diabetes and the prevalence of islet amyloid. The high prevalence of amyloid deposits in diabetic patients treated with insulin and the low presence of amyloid in non-diabetics indicates that endogenous insulin reaction plays an essential role in the development of amyloid. Moreover, a multicenter study aimed to estimate the prevalence of amyloid deposits in a group of patients with and without diabetes. Patients whose autopsy was performed within the first 24 h of death were included in the study. Data regarding the plasma glucose levels, hemoglobin A1C, total cholesterol, triglyceride, low-density lipoproteins, high-density lipoproteins, body mass index, and the sufficiently harvested pancreatic tissue were available. The presence of amyloid deposits was identified as being higher (39.6%) in patients with diabetes than in non-diabetic patients (3%). In diabetic patients the presence of amyloid was associated with a higher body mass index and poor glycemic control. The study showed a higher frequency of pancreatic fibrosis and fat infiltration in diabetic patients with amyloid deposits compared to those without amyloid. Pancreatic atherosclerosis was identified in all diabetic patients enrolled in the study [53]. Studies have shown that the formation of amyloid deposits in beta cells is a diabetogenic factor and that there is a close relationship between them and the development of diabetes. However, the role of island amyloid in diabetes is still being debated. There is no certainty whether the island amyloid is directly involved, or it is just the consequence of the disease. It is also unknown if diabetic hyperglycemia and insulin resistance induce amyloid deposition in the islets and brain [54].

Using brain samples from the temporal cortex from patients confirmed with AD at autopsy and from non-AD patients, researchers investigated the simultaneous presence of IAPP and amyloid beta in the human brain. Western blot analysis showed that samples from AD patients contained 1.4 times higher concentrations of IAPP than non-AD samples. Beta amyloid was detected in higher concentrations in AD samples than in non-AD samples [55]. Jackson et al. [56] investigated brain samples from patients with diabetes and dementia (vascular dementia or AD), patients with AD and no history of diabetes, and samples from healthy people. The immunohistochemically examination of the gray matter of the temporal lobe from diabetic patients with dementia revealed many amylin plaques. Amylin deposits were identified in brain samples from patients with AD and no diabetes. In these samples, the distribution of amylin deposits was like that of the group of samples from patients with diabetes mellitus and dementia. In the control group, rare amylin deposits were found in the brain parenchyma and in blood vessels. Pancreatic amylin is being accumulated in the brain as independent plaques or its co-primes with beta amyloid to form complex amyloid-beta amyloid plaques. The latter contributes to the neurodegeneration of the patient with diabetes mellitus. In a clinical study, Janson et al. [34] investigated the prevalence of diabetes in patients with AD and in patients without AD. The prevalence of diabetes was higher in patients with AD versus the control group (34.6% vs. 18.1%). In parallel, the authors performed a pathological study on brain and pancreas samples. The frequency and the extent of amyloid were higher in the AD group compared to the non-AD control group. Amyloid deposits were also higher in patients with diabetes compared with the non-diabetes group. No differences were found regarding the density of diffuse or neuritic plaques in the patients with diabetes compared with the control group non-diabetics. 

Insulin is the major gluco-regulator of energy homeostasis as well as a modulator of brain activity, a hormone that enhances glucose uptake and metabolism in neurons and glial cells [57]. From a physiological point of view, insulin binds to insulin receptors (IR), triggering the phosphorylation of the insulin receptor substrate (IRS1) involved in the activation of phosphatidylinositol 3 kinase (PI3K) at the brain level by modulating synaptic plasticity and cognition, with neurotrophic, neuro-modulatory, and neuroprotective effects [58,59].

Among the common features of AD and diabetes, an etiological factor in AD is the reduced cellular reaction to insulin, a phenomenon highlighted in a post-mortem study when the causes and consequences of insulin resistance at the brain level were analyzed, especially regarding the hippocampus, the dentate gyrus, and the subiculum, favoring the emergence of a marked early pathology, with this insulin resistance of the brain being a frequent feature in AD patients [60]. Insulin reaches the brain through the blood–brain barrier (BBB) to take part in various cognitive processes. Insulin synthesis and secretion play an important role in maintaining glucose levels within normal limits. In this regard, some researchers have observed that glucose uptake and metabolism in the brain are regulated by insulin, and its imbalances causes the disruption of energy metabolism with the production of reactive oxygen species (ROS) and mitochondrial dysfunction, followed by activation of the pro-apoptotic and inflammatory cascade, Aβ-forming APP cleavage [61]. Moderate insulin concentrations at the brain level have neurotrophic properties, and elevated concentrations are associated with the reduced amyloid clearance [62]. The brain insulin deficiency in AD appears to be mediated by the activity of two enzymes: the insulin-degrading enzyme (IDE) and the calcineurin 1 regulator (RCAN1). The fact that IDE may degrade insulin, amylin, and Aβ suggests that AD may be linked to diabetes via chromosome 10 and the finding that Aβ plaque size correlates inversely with IDE expansion and activity suggests that IDE deficiency may mediate plaque accumulation and cognitive impairment in AD [63]. The concept of insulin resistance was described in 1940 and since then a clear picture has emerged about its involvement in AD. Moreover, insulin resistance, and lipid and carbohydrate metabolism lead to Aβ aggregation and memory impairment [51,64]. Cognitive defects in diabetes are associated with insulin resistance that affects psychomotor efficiency, attention, learning memory, mental flexibility, speed, and executive function of the brain. The basic pathophysiology of cognitive dysfunction in diabetes is not fully known and characterized. However, in recent years, several hypotheses have been proposed and research results have been published that validate such hypotheses. The theories underlying cognitive dysfunction are usually correlated with hyperglycaemia, vascular disease, hypoglycaemic episodes, insulin resistance, and amyloid [65,66]. Defective insulin secretion by pancreatic β-cells and insulin sensitivity are the main causes of diabetes mellitus type 2. In addition to peripheral insulin function, it also intervenes in the regulation of synaptic and neuronal function in the cortex, cerebellum, and hippocampus, as well as in the protection of neurons from brain death. It also acts on the enzyme for the elimination of β-site precursor protein (BACE1) and γ-secretase to regulate Aβ levels and degrade excess Aβ by modulating insulin-degrading enzyme [51]. Another study (Hisayama study) conducted between 1998–2003 in patients of both sexes suggested that histopathological features present in AD are associated with diabetes or insulin resistance on the basis of the relevant statistical data that have shown that hyperglycaemia and hyperinsulinemia are major risk factors in the development of amyloid plaques [67]. Insulin resistance appears due to the reduced ability of the insulin receptor to respond to insulin stimulation, this resistance being a major feature of diabetes that could be detected long before the clinical signs of the disease [68]. There is evidence to support the fact that, with age, insulin levels increase, and that hyperinsulinemia is associated with a functional decline in the central nervous system (CNS). Some studies in older men have reported that men with the highest insulin levels, when performing the Mini-Mental State test, had 25% more errors compared to those with very low insulin levels [69]. The accumulation of amylin in the pancreas can decrease the level of insulin, which will lead to disruptions in carbohydrate metabolism (hyperglycemia) and will promote the development of neurodegenerative disorders [70]. The influence of high glucose levels on cognitive impairment was highlighted in the ACCORD-MIND study, which by assessing glycosylated hemoglobin (HbA1c) could detect the negative impact on cognitive tests [71]. It is possible that chronic exposure of the brain to high glucose levels may hasten cognitive decline, which was observed in post-mortem studies carried out with patients with AD where amyloid plaques and metabolic degradation products associated with hyperglycemia were found [72,73]. Hyperglycemia levels most likely due to poor insulin synthesis could be corrected by using mediational therapies involved in increasing insulin levels. There are many clinical trials in AD patients who support these assertions and have shown that medication used to increase insulin levels has improved the condition of patients with cognitive impairments [74]. Recently, more and more attention has been devoted to the role played by glycation of proteins in promoting amyloid aggregation and cell toxicity [75]. In hyperglycemic conditions, proteins are highly susceptible to non-enzymatic glycation, and this post-translational modification could differentially affect the aggregation process and amyloid formation in promoting, accelerating, and/or stabilizing on-pathway and off-pathway species [76]. The observation, according to which proteins from amyloid deposits such as Aβ-peptide, IAPP, tau, prions, and transthyretin can be glycated, indicates a direct relationship between protein glycation and amyloidosis [77]. Thus, in vitro and in vivo studies have shown that non-enzymatic glycation promotes the formation of amyloid fibrils in IAPP [78] and may stabilize the toxic oligomeric species related to neurotoxicity for both Aβ-peptide [79] and human insulin [80]. Glycation is a pathological process implicated in the chronic complications of diabetes and has been described to play a crucial role in normal aging process and pathogenesis of Alzheimer’s disease [77,81].

## 3. The Influence of Amyloid-β Aggregates on Diabetes Pathology and Islet Amyloid Polypeptide on Alzheimer’s Disease in Animal Models

The relevance of exploring these two amyloids may not be stressed enough given that pancreatic IAPP aggregation has been found in over 96% of autopsied diabetes individuals [82,83], and Aβ plaques are constantly reported in the brain of the AD patients [17,84]. Cerebral insulin resistance has been highlighted to intensify Aβ fibrillogenesis by increasing and clustering of GM1 ganglioside in the neuronal membranes [85] and cognitive decline [86]. In both humans and rodents affected by early AD, insulin administration has been correlated with reduced amyloid accumulation and improved cognitive performance [87,88]. Preclinical induction of diabetes has been able to exacerbate tau pathology [89,90]. In addition, in murine models of AD, chronic anti-diabetic therapy may reduce tau deposits and the β-amyloid plaque count by 40%–50% [91], and rescue spatial memory and recognition impairments [92]. Mostly in diabetic cases, low glucose brain metabolism, a negative effect of insulin deficiency or insulin resistance, could be the main culprit for the risk intensification of AD development.

Most clinical studies link diabetes to AD, and diabetics have a twofold increased risk of developing AD [82,93,94], while another part of the studies suggest an inverse relationship [34]. Synthetic preformed fibrils of human IAPP 1 to 37, or Aβ 1 to 42 (corresponding to 20 μg of peptide in 100 mL of 0.15 mol/L sodium chloride) intravenously injected into tail vein of human IAPP transgenic mice acted as a seed for IAPP in the islet of Langerhans. The findings showed not only a higher number of mice with islet amyloid, but also the number of islets with amyloid was substantially increased from 2.7% and 5% in animals inoculated with sodium chloride and human des−31,32 proinsulin, respectively, to 24.1% in individuals injected with human IAPP fibrils, and 15.2% in those with Aβ fibrils. Morphological evaluation using proximity ligation assay and Western blot analysis of IAPP and Aβ in islets of diabetics and cerebral Aβ deposits of AD cases revealed co-localization of IAPP and Aβ in brain and cerebrovascular amyloid deposits, while Aβ reactivity was not identified in any amyloid aggregates of endocrine pancreas. In this study it could not be identified if the IAPP found in the brain was locally formed or derived from pancreatic B cells. Taken together, the heterologous seeding between IAPP and Aβ highlighted here may support a molecular link between these chronic amyloidogenic conditions [55].

Previous studies have shown that inoculation of AD brain tissue extracts could be used for experimental induction of Aβ plaques and cerebral beta-amyloid angiopathy in non-human primates [95] and transgenic APP mice [96,97], although inoculation of preformed purified and synthetic fibrils from Aβ40/42 was less effective [98,99]. The detection of multiple mouse and human Aβ42/40 variants in cerebral amyloid deposits formed in human APP-London transgenic mice offered evidence that APP mice may be processed into Aβ [100]. Therefore, it can conclude that the conditions required for Aβ fibrillation are found in transgenic IAPP mice, but despite this, mice inoculated with Aβ fibrils developed only IAPP amyloid in the islet of Langerhans. In an attempt to clarify the mechanism of cerebral beta-amyloid induction by intraperitoneal administration of Aβ-comprising brain extracts in preclinical models, Eisele et al. [101] used three APP transgenic mouse strains that vary in levels of transgene expression in the brain (APP23 mice and APP23 mice missing murine APP) and also to some extent in systemic organs (R1.40 mice). Remarkably, two intraperitoneal injections of 100 μL of brain extracts resulted in an earlier and strong induction of cerebral amyloid in APP23 strain than in R1.40 host. These data are consistent with a nearly five- to sevenfold overexpression of human APP in the brain of APP23 mice [102] compared to the threefold overexpression of human APP in the R1.40 mice [103]. Despite a peripheral expression of Aβ-precursor protein, R1.40 mice never developed beta-amyloid outside the brain. These findings reflected a more complex mechanism for Aβ seeding, and the lack of Aβ deposited in islet amyloid may be directly dependent on low or no Aβ production in peripheral tissue.

To elucidate the pathophysiological relationship between diabetes and AD, Takeda and co-workers [104] developed two mouse models that reveal the pathological aspects of both conditions. They crossed a murine model of AD (APP23) with two strains of diabetic mice (ob/ob and Nagoya–Shibata–Yasuda (NSY) mice) and explored their brain and metabolic pathology. In double transgenic APP × ob/ob mice, the onset of diabetes caused an exacerbation of Alzheimer-like cognitive impairment, without an increase of the Aβ load in the brain, supporting the notion that Alzheimer pathology could promote diabetes. APP × NSY fusion mice displayed marked glucose intolerance compared to their control groups. Notably, in both mouse models, these findings were associated with cerebrovascular inflammation and an important brain amyloid angiopathy. However, this research did not fully establish whether AD could accelerate the development of the diabetic phenotype. 

Over time, epidemiological, clinical and basic research has revealed a direct relationship between diabetes and AD. To test the hypothesis that AD would determine the onset of diabetes, Jiménez-Palomares et al. [105] generated a new transgenic mouse model. Thus, they cross-linked transgenic AD APPswe/PS1dE9 mice with (db/+) mice, partially deficient in leptin signaling, and monitored their body weight, and insulin and plasma glucose levels. Phenotypic characterization of glucose metabolism was performed using glucose and insulin tolerance tests. Histomorphometry was used to analyze the cell mass, number of islets, and their volume. Compared with the control group (APP/PS1 mice with co-expression in mice with intact leptin receptor signaling), the novel developed mouse model (APPswe/PS1dE9 × db/+) showed noneating hyperglycemia, hypercholesterolemia, and hyperinsulinemia, with no alterations in body weight. In contrast, fasting glucose homeostasis and cholesterol levels remained unchanged between APP/PS1 co-expression in db/+ mice and their control. Concurrent with modified glucose metabolism, APPswe/PS1dE9 × db/+ mice led to glucose intolerance, insulin resistance, and altered insulin signaling. These results were paralleled with an increase in β-cell mass expansion, providing at the same time experimental evidence that supports the idea that abnormal Aβ production could be a mechanistic link that underlies pathology of insulin resistance and diabetes in Alzheimer’s disease. 

To more fully clarify neuro-inflammatory alterations related with diabetes that could drive AD pathology, Sankar and co-workers [70] quantified cortical modifications in cytokine proteins in three different models of mice, with metabolic changes relevant to diabetes combined with a mouse model of AD (APP/PS1). Multiplexed immunoassay showed that pathology associated with either pre-diabetes, db/db, or streptozotocin models led to the upregulation of a comprehensive profile of cytokines, comprising chemokines (macrophage inflammatory protein-1 alpha, MIP−1α; monocyte chemoattractant protein-1, MCP−1; and macrophage inflammatory protein-1 beta, MIP−1β) and proinflammatory cytokines (IL−1α; interferon gamma, IFN-γ; and IL−3). Moreover, the APP/PS1 × db/db mice model presented circulating levels of Aβ40/42, glucose, and insulin correlated with brain cytokine expression, supporting a robust relationship between peripheral alterations and cerebral pathology. In this context, the authors suggest that since most of the highlighted cytokines stimulate neuronal damage, tau, and Aβ pathology, as well as breakdown of the blood–brain barrier, neuro-inflammation may modulate the effects of diabetes on the pathogenesis of AD. 

In translational research, the casual or causal relationship between diabetes and AD in the past decade has been intensively studied in murine AD models on hyperlipidemia polygenic backgrounds, intake of ultrahigh-fat diets [104,105,106,107,108], or streptozotocin-induced b-cell death acute models [109,110]. However, these models due to the incapacity of rodents to aggregate IAPP did not reveal amyloid IAPP deposition. To study the interconnection between these amyloidogenic diseases, animal models have been generated that co-express both human Aβ and IAPP pathologies, able to closely resemble the clinical presentation of diabetes, permitting a more precise replication of insulin-signaling defects that appears in humans. Apolipoprotein E (ApoE), a constituent of the deposits, has been identified in the islet cells as having a significant role in disorders of lipid metabolism, which are often related with diabetes [111,112]. In addition, the most common occurring isoform, ApoEO4, has been involved in plaque formation in patients with AD. However, no association has been found between ApoE genotype and the degree of islet amyloidosis in post-mortem specimens or with severity of diabetes [113]. An association between diabetes, ApoE genotype, and dementia has been identified in Polynesian/American men, but the degree of islet amyloidosis was not determined [93]. Studies by Wijesekara et al. [114] have shown that Aβ and IAPP are crucial features in the overlapping pathologies of diabetes and AD. When they crossed AD-related human APP transgenic mice to human IAPP homozygote animals, this study found that the novel double transgenic mouse model was markedly hyperglycemic, exhibiting severe insulin resistance and glucose intolerance, accompanied by exacerbated brain pathology. IAPP and Aβ amyloid co-deposition significantly elevated tau phosphorylation, and a decrease in pancreatic b-cell mass was detected in islets. In 24-week-old mice, both increased tau phosphorylation, plaque load, and hippocampal total Aβ42 levels, and decreased insulin levels and signaling were complemented by widespread synaptic loss and reduced neuronal counts. In addition, synthetic Aβ42 immunization to verify whether peripheral Aβ contributes to insulin resistance has been associated with the rescue of hyperglycemia and peripheral insulin resistance, suggesting an important role for Aβ in the pathogenesis of diabetes for people predisposed to AD. 

The precise relationship between amyloid peptides is not currently known, but it is highly likely that formation and accumulation of one protein may promote the misfolding and aggregation of another one, indicating a possible cross-seeding effect. Misfolded IAPP can be formed and deposited in the brain of diabetes and AD patients [56], whereas Aβ and tau proteins can be detected in pancreatic islets in diabetes [83]. The effect of the co-existence of both IAPP and Aβ in vivo and the intensification of brain amyloid deposition by exogenous use of IAPP aggregates in transgenic AD mouse models was investigated by Moreno-Gonzalez et al. [115]. The authors generated and characterized models of double transgenic mice named IAPP^+/−^ × APP and IAPP^+/+^ × APP, comprising one copy of mutant human APP gene (APPSwe^+/−^) and one (IAPP^+/−^) or two (IAPP^+/+^) human IAPP copies. Transgenic mice expressing both IAPP and Aβ proteins displayed an exacerbation of both plaque density and Aβ burden in the brain as opposed to transgenic AD animals or transgenic AD mice with type−1 diabetes. Interestingly, in the parenchymal deposits of the brain, IAPP polypeptide co-localized with amyloid plaques, suggesting that these peptides can interact directly, aggravating the disease. Similar results were also observed in transgenic rats overexpressing human IAPP in the pancreas, which also showed IAPP deposits in the cerebral parenchyma [116]. In addition, pancreatic tissue examination from mice over-expressing the mutant human APP gene revealed a substantially higher IAPP load, signifying that Aβ pathology may also stimulate pancreatic IAPP aggregation. Furthermore, intracerebral injection of aggregated IAPP homogenates into the APP mice brains led to a more intense AD pathology accompanied by severe memory deficits than untreated animals. Collectively, these data suggest that IAPP and Aβ may interrelate by cross-seeding, offering a new potential explanation for the higher risk of AD in people affected by diabetes [115]. In addition, the results of Moreno-Gonzalez et al. [115] are supported by an earlier observation in non-human primates that spontaneously develop both AD and diabetes conditions, which presented accelerated Aβ pathology compared to non-diabetic monkeys [117]. This research supports the conclusion that the coexistence of Aβ and IAPP in the same patient and the distinctive features of these amyloid peptides contribute to accelerate or increase disease phenotypes. 

## 4. Relevance of Molecular Interaction between Islet Amyloid Polypeptide and Amyloid-β Peptide for Novel Therapeutics

Given the poor epidemiological forecast, research policies, and strategies, as well as the large body of reports that diabetes and associated traits increase the risk for developing AD, there has been an extraordinary interest in exploring whether the use of antidiabetic compounds could impact the risk of dementia, and whether these drugs may be utilized to prevent or treat Alzheimer’s disease. Thus, dozens of studies have been undertaken to evaluate the extent to which antidiabetic treatments could impact brain pathology (Table 2), especially AD features, with the most part of studies targeting potential benefits on amyloid pathology [118,119], cognitive function [120,121,122], tau pathology [123,124], neuroinflammation [125], oxidative stress [126], neurogenesis [127], and synapses [128,129]. 

Although it has been highlighted that self-assembly of amyloid is related to pathogenesis of diabetes and AD, to date, no amyloid inhibitor compounds have touched the clinic. This fact is in particulr due to the high conformational flexibility of the majority of amyloidogenic peptides, the interactions with high affinity between amyloid self-assembly, and the generous size of interfaces implicated. Moreover, supplementary challenges comprise the low BBB permeability, higher costs of production, possible immunogenicity of antibodies, and differential proteolytic stability [162]. Instead, macrocyclic peptides might be a reasonable alternative, because they most often combine highly promising “drug like properties”, such as a great surface area, increase binding affinity, target selectivity, and high activity, improving the stability and cell permeability in biological fluids. Thus, a fairly large number of Aβ40/42 peptide-based inhibitors or IAPP amyloidogenesis were derived from their target polypeptides [163,164,165]. Therefore, Spanopoulou et al. [166] designed macrocyclic peptides using IAPP-derived minimal recognition components as a new class of amyloid inhibitors of both IAPP and Aβ40/42, or Aβ40/42 alone, and revealed that chirality controls inhibitor selectivity. In addition, sequence optimization performed led to the detection of Aβ40/42 selective macrocyclic peptides, with advanced proteolytic stability and human plasma and BBB crossing capacity, in vitro. Given to their promising properties, these compounds could aid as factors for macrocyclic peptide on the basis of anti-amyloid agents and scaffolds for the design and development molecule for directing amyloidogenesis in both diabetes and AD. 

In vitro and in vivo exploration of molecular interaction between IAPP and Aβ through self-/cross-interaction of protein misfolding can explain at least in part why there is a coexistence of the two amyloidogenic proteins in a single patient with a higher prevalence, as well as the augmented clinical features in some cases. Moreover, self-/cross-interaction mechanisms may explain why a part of conditions affiliated with protein formation and deposition are localized in aberrant regions of the affected body (e.g., IAPP co-localization with Aβ in the pancreas and Aβ-IAPP co-localizing in the brain). The fact that IAPP and Aβ are amyloids with common structural features permits the development and validation of new therapeutic approaches that target both proteins, offering more effective compounds. Additional data on the synergism of these conditions will likely alleviate the number of people affected by both diabetes and AD, lowering the prevalence of both diseases and moreover ameliorating the economic burden of these chronic maladies in the world.

## 5. Concluding Remarks

As the life expectancy of the population constantly increases along with the number of people living unhealthy lifestyles, the prevalence of age-related diseases, such as diabetes and AD, also increase. Thus, it is estimated that by 2050, the prevalence of these conditions will double or even triple. Although these statistics are at present quite unpleasant, current studies suggest that the existence of diabetes in a patient can increase the risk of AD by 2–5-fold, and the number of diabetics among the AD population is considerably increased in comparison to non-AD group controls that have been age-matched. Exploring further the role of molecular interaction between islet amyloid polypeptide and amyloid-β peptide, two amyloidogenic proteins, as a common pathological feature in both diabetes and Alzheimer’s disease, may offer novel directions in biomarker development and innovative therapeutic interventions that can prevent or reduce the occurrence of diabetes, as well as AD. Since the pathological aspects attributable to these amyloidogenic proteins involve a wide range of organ systems usually related with diverse disciplines, multidisciplinary approaches by researchers and clinicians with different expertise and interests would be essential to move the field forward. All these efforts promise to promote our understanding of an understudied but crucially aspect of the pathobiology of diabetes in relation to AD.

## Figures and Tables

**Table 1:** Current therapies in diabetes and Alzheimer’s disease.

**Diabetes**	Insulin
	Biguanides: Metformin
	Sulphonylureas: Glibenclamide, Glibornuride, Glipizide, Gliquidone, Glisoxepide, Glyclopyramide, Glimepiride
	Alpha-glucosidase inhibitors: Acarbose, Miglitol, Voglibose
	Incretins Dipeptidyl peptidase−4 inhibitors: Sitagliptin, Saxagliptin, Linagliptin, Alogliptin Glucagon-like peptide−1 receptor agonists: Exenatide, Liraglutide, Albiglutide, Dulaglutide
	Thiazolidinediones: Pioglitazone, Rosiglitazone
	SGLT2 Inhibitors: Empagliflozin, Canagliflozin, Dapagliflozin, Ipragliflozin
	Meglitinides: Repaglinide, Nateglinide
	Amylin analog: Pramlintide
**Alzheimer’s Disease**	Cholinesterase inhibitors: Tacrine, Donepezil, Rivastigmine, Galantamine
	N-methyl-D-aspartate receptor: Memantine

**Table 2:** Comparative efficiency and acceptability of antidiabetic compounds for Alzheimer’s disease.

Antidiabetic Medication	Experimental Model	Findings	References
Biguanides			
Metformin	mouse neuroblastoma cell lines under sustained hyperinsulinemic conditions treated with different concentrations of metformin (0.4–3.2 mM)	resensitization of insulin signaling; prevention of the molecular and pathological alterations detected in AD neurons	[130]
	transgenic APPswe/PSd1E9 mouse model of AD; intraperitoneal delivery of 200 mg/kg metformin for 14 days	amelioration of spatial memory deficits, neural cellular proliferation; in the cortex and hippocampus, reduction of local inflammation, decrease of Aβ plaque deposition	[119]
	PDAPP (J9) mouse model of AD; 350 mg/kg/day metformin delivered in drinking water for several months	attenuation of memory impairment in female subjects and intensification of it in males	[131]
	longitudinal aging study in adults with diabetes	long-term metformin therapy (over 6 years) could diminish the risk of developing AD	[132]
	case-control study, older adults with an incident diagnosis of AD; 1–9, 10–29, 30–59, or ≥60 metformin prescriptions	long-term treatment (60 or more prescriptions) has been correlated with a slight augmented risk of developing AD	[133]
Sulphonylureas			
Glibenclamide	Aβ25–35-induced rat AD model; 6 mg/kg/day of glibenclamide for 20 days by gavage	reduction of Aβ25–35-treated behavioral anomalies	[134]
Thiazolidinediones			
Pioglitazone	meta-analysis of randomized clinical trials; 15 to 30 mg of pioglitazone, as adjunct therapy for AD	doses of 15 to 30 mg pioglitazone but not 45 mg improve cognitive capacity	[135]
	transgenic APPswe/PSEN1dE9 AD mouse model; combined therapy with 0.03 mg/kg/day of leptin intranasal delivery + intraperitoneal administration of 10 mg/kg/day pioglitazone for 2 weeks	decrease of spatial memory impairments and brain Aβ levels	[122]
	APPV717I transgenic mice, a model for AD; acute 7 days gavage therapy with 40 mg/kg/day of pioglitazone	reduction of soluble Aβ1–42 peptide levels by 27% and glial inflammation	[125]
	controlled trial in cases with mild Alzheimer’s disease and an accompanying diagnosis of diabetes; daily doses of 15–30 mg pioglitazone for 6 months	cognitive and functional improvements and stabilization of the disease in diabetics with AD	[136]
	controlled pilot trial in individuals with AD without diabetes; daily 45 mg of pioglitazone	18 months of pioglitazone therapy were well tolerated by patients, but no important efficacy data were detected	[137]
Rosiglitazone	meta-analysis of randomized clinical trials; 2 to 8 mg of rosiglitazone, as adjunct therapy for mild to moderate AD patients	pro-cognitive effects	[135]
	pilot study that randomized individuals with AD or amnestic mild cognitive damage	better delayed recall and selective attention	[138]
	large study in population with mild to moderate AD; 2, 4, or 8 mg of rosiglitazone for 6 months	in week 24 an improvement (−2.9 points) of cognition in apolipoprotein Eε4-negative people treated with 8 mg of rosiglitazone was registered	[139]
	phase III trials of rosiglitazone in AD; 2 mg or 8 mg rosiglitazone for 48 weeks, as adjunctive agent to ongoing acetylcholine esterase inhibitors	rosiglitazone did not lead to an improvement in cognition or overall function	[140]
Glucagon-like peptide−1 receptor agonists			
Lixisenatide	transgenic APPswe/PSd1E9 mouse model of AD; intraperitoneal injection with 1 or 10 nmol/kg of compound for 10 weeks	several biomarkers have been improved such as learning, inflammation, or plate loading	[129]
	cell culture, 100μM of lixisenatide were applied 24 h before Aβ25–35 application rat model of AD; 5 nmol/μL of lixisenatide before intrahippocampal application of Aβ25–35 (5 nmol/μL)	reversal Aβ25–35-triggered cytotoxicity, normalization of intracellular calcium levels prevention of memory loss caused by amyloid intracerebroventricular injection	[141]
	transgenic APP/PS1/tau mouse model of AD; daily intraperitoneal injection of 10 nmol/kg lixisenatide for 60 days	reduction of amyloid plaques, neuroinflammation, and neurofibrillary tangles	[142]
Dulaglutide	intracerebral injection of streptozotocin-induced mouse AD-like condition; 0.6 mg/kg/week of dulaglutide with intraperitoneal delivery for 4 weeks	amelioration of learning and memory deficits	[143]
Liraglutide	transgenic APPswe/PSd1E9 mouse model of AD; intraperitoneal injection with 2.5 or 25 nmol/kg of drug for 10 weeks	improvement of learning, reduction of amyloid plaque deposits by 40%–50%, and decrease inflammatory response	[129]
	methylglyoxal-induced mouse Alzheimer-like condition; daily subcutaneous administration of 25 nmol/kg liraglutide for 2 months	attenuation of hippocampal damage and cognitive deficits in C57BL/6J mice	[144]
	cell culture; liraglutide (300 nm) was added to cultures 40 min before Aβ oligomers Aβ oligomers-induced AD mouse model; daily intraperitoneal injections of liraglutide (25 nmol/kg) for 7 days Aβ oligomer-induced non-human primate model of AD; subcutaneous delivery of liraglutide (0.006 mg/kg/day for the first week and 0.012 mg/kg thereafter) for 24 days	reduction of Aβ oligomer-induced synaptotoxicity, protective effects on synapses; prevention and reversal of cognitive abnormalities, and insulin receptor loss produced by intracerebroventricular injection of Aβ oligomers; the agent was less effective, but still provided partial protection against insulin resistance loss; synapses and phosphorylation of tau	[121]
	Aβ protein-induced rat model of AD; 2 μL liraglutide trough intrahippocampal administration	liraglutide pre-therapy remarkably protected against Aβ-induced damage of spatial memory and long-term potentiation	[145]
	transgenic 3xTg-AD female mice; 0.2 mg/kg/day of liraglutide, intraperitoneal injections	reduction of cortical Aβ1–42 levels, partial attenuation of cerebral estradiol, inflammation, and oxidative/nitrosative stress	[146]
	a pilot clinical trial in AD patients lasting 26 weeks; in the first week, the drug was daily delivered subcutaneously at a dose of 0.6 mg; hereafter 1.2 mg daily for another week before finally increasing to 1.8 mg daily	prevention of brain glucose metabolism decline; there were no important cognitive changes compared with placebo group	[147]
Dipeptidyl peptidase−4 inhibitors			
Saxagliptin	intracerebral injection of streptozotocin-induced rat model of AD; 0.25, 0.5, and 1 mg/kg of saxagliptin administered orally for 60 days	reduction of amyloid plaque formation, a marked decrease of Aβ42 level, and phosphorylation of tau protein; total reversal of cognitive impairments	[148]
Vildagliptin	intracerebral injection of streptozotocin-induced rat model of AD; daily oral doses of 2.5, 5, and 10 mg/kg vildagliptin for 30 days	attenuation of Aβ, phosphorylation of tau protein, and inflammatory markers	[149]
	Aβ protein-induced rat model of AD; daily gavage of 5 or 10 mg/kg vildagliptin for 4 weeks	anti-apoptotic effect, attenuation of memory abnormalities, reduction of tau phosphorylation, and increase of neurotrophic protein expression	[150]
	streptozotocin-induced rat diabetes model associated cognitive decline; daily gavage of 5 mg/kg vildagliptin for 4 weeks	prevention of memory impairment and diminution of apoptosis in hippocampal neurons	[151]
Sitagliptin	APP/PS1 AD mice model; 20 mg/kg/day of sitagliptin for an 8-weeks period	protective effect of cognitive function, reduction of amyloid plaque deposits	[152]
	transgenic APPswe/PSd1E9 mouse model of AD; daily gavage of 5, 10, and 20 mg/kg sitagliptin for 12 weeks	much more obvious effects for the 20 mg/kg sitagliptin dose—reduction of nitrosative stress and inflammation markers; an important diminution in the number and area of APP and Aβ deposition	[153]
Linagliptin	3xTg-AD mouse model of AD; daily oral administration of 5, 10, and 20 mg/kg linagliptin for 8 weeks	improvement of cognitive performance; reduction of Aβ42 levels, but not Aβ40; diminution of tau phosphorylation and neuroinflammation	[154]
	human neuroblastoma SK-N-MC cell culture; exposure to 10 to 100 μM linagliptin for 24 h	protection of cells against Aβ-induced intracellular reactive oxygen species accumulation and mitochondria dysfunction	[155]
Amylin analog			
Pramlintide	SAMP8 mice, a model of sporadic AD; subcutaneous infusion of 0.24 mg/kg/day pramlintide for 5 weeks	may improve memory, decrease neuroinflammation, and reduce oxidative stress	[156]
Sodium-glucose cotransporter 2 (SGLT−2) inhibitors			
Canagliflozin	scopolamine-induced rat model of memory impairment; daily oral gavage of 10 mg/kg for 2 weeks	improvement of memory dysfunction	[157]
Insulin analogues			
	intracerebral injection of streptozotocin rat model of cognitive decline; 0.5 units = 12 nmol of detemir	alleviating cognitive dysfunction with a significant increase in learning ability; change in insulin degrading enzyme, insulin receptor, and somatostatin	[158]
	patients with early AD; intranasal administration of 20 or 40 IU insulin	facilitation of verbal memory recall in memory-impaired ɛ4− patients; no influence on glucose or plasma insulin levels	[159]
	patients with early AD; intranasal administration of 20 or 40 IU insulin for 21 days	improvement of attention, functional status, and verbal memory; modulation of Aβ peptide	[87]
	placebo-controlled pilot clinical trial in people with AD; intranasal delivery of 20 or 40 IU insulin for 4 months	improvement of cognition and functional ability compared to control group	[160]
	clinical trial; 20 or 40 IU of insulin detemir for 21 days, intranasal administration in AD	therapy effect for the memory composite outcome for the 40 IU patients, influenced by the APOE status	[161]

## Abbreviations

AD, Alzheimer’s disease; Aβ, amyloid-β peptide; IAPP, islet amyloid polypeptide; APP, amyloid precursor protein; ADDLs, diffusible Aβ derivatives or protofibrils; PSEN−1, presenilin−1; PSEN−2, presenilin−2; GM1, GM1 ganglioside; NSY mice, Nagoya–Shibata–Yasuda mice; AICD, cytoplasmic polypeptide; BACE1, beta-secretase 1; APPswe/PS1dE9 mice, APP/PS1 double transgenic mouse model of Alzheimer’s disease over expressing amyloid precursor protein (APPswe), encoding the Swedish mutations at amino acids 595/596 and an exon−9-deleted human PS1 (PS1dE9); APPV717I mice, transgene containing human APP (isoform 695) with the London mutation as model for Alzheimer’s disease and cerebral amyloid angiopathy; SAMP8 mice, senescence-accelerated prone mouse, a mouse model of sporadic AD; 3xTg-AD mice, triple transgenic mouse model of Alzheimer’s disease; SGLT−2, sodium-glucose cotransporter 2 inhibitors; MRI, magnetic resonance imaging; IR, insulin receptors; IRS1, insulin receptor substrate; PI3K, phosphatidylinositol 3 kinase; ROS, reactive oxygen species; IDE, insulin-degrading enzyme; RCAN1, calcineurin 1 regulator; BBB, blood–brain barrier; ApoE, apolipoprotein E.
